# Supplementary material for: Identifying and characterising a plant GH1 β‐glucosidase that exhibits hydrolytic activity on N‐linked glucopyranoside
Source: FEBS Open Bio. 2026 Aug 6:10.1002/2211-5463.70309. Online ahead of print. doi: 10.1002/2211-5463.70309 (PMC13447983; doi:10.1002/2211-5463.70309)
Supplement: Supplementary file 1 [file FEB4-9999-0-s001.docx]

**Discovery and Characterisation of a Plant GH1 β-Glucosidase Exhibiting Hydrolytic Activity on an *N*-Linked Glycopyranoside**

Hani Gharabli^1^, Carlotta Chiesa^1,2^, Maher Abou Hachem^3^, Ditte Hededam Welner^1*^

^1^The Novo Nordisk Center for Biosustainability, Technical University of Denmark, Kongens Lyngby, Denmark

^2^Department of Chemistry, Materials and Chemical Engineering “Giulio Natta”, Politecnico di Milano, p.zza L. da Vinci 32, Milano, Italy

^3^Department of Biotechnology and Biomedicine, Technical University of Denmark, Kongens Lyngby, Denmark

^*^Corresponding author, diwel@biosustain.dtu.dk

The supplementary information includes:

**Supplementary Tables**

Supplementary Table 1. Bgl library

**Supplementary Figures**

Supplementary Figure 1. *In vivo* degradation of MANT-*N*-glucose

Supplementary Figure 2. *In vitro* degradation of MANT-*N*-glucose

Supplementary Figure 3. Chromatograms of the *Zm*Glu1-catalysed hydrolysis

Supplementary Figure 4. *Zm*Glu1:MANT-*N*-glucose complex structures

# **Supplementary Tables**

| Bgl | PDB | UniProt accession | Organism | Reference |  |
| --- | --- | --- | --- | --- | --- |
| *Hi*Bgl4 | 4MDO | O93784 | *Humicola insolens* | [19] |  |
| *Hs*KLrP | 2E9L | Q9H227 | *Homo sapiens* | [20] |  |
| *Nk*Bgl | 3AHZ | Q8T0W7 | *Neotermes koshunensis* | [21] |  |
| *No*BGLN1 | 5YJ7 | A0A452CSM4 | *Nannochloropsis oceanica* | [22] |  |
| *Os*3BGlu6 | 3GNO | Q8L7J2 | *Oryza sativa* | [23] |  |
| *Os*4BGlu12 | 3PTK | B8AVF0 | *Oryza sativa* | [24] |  |
| *Os*7BGlu26 | 4JHO | A2YPH1 | *Oryza sativa* | [25] |  |
| *Os*BGlu1 | 2RGL | Q75I93 | *Oryza sativa* | [26] |  |
| *Pc*BGL1A | 2E3Z | Q25BW5 | *Phanerodontia chrysosporium* | [27] |  |
| *Rs*RG | 4ATL | Q9SPP9 | *Rauvolfia serpentina* | [28] |  |
| *Rs*SG | 2JF7 | Q8GU20 | *Rauvolfia serpentina* | [29] |  |
| *Sb*Dhr1 | 1V02 | Q41290 | *Sorghum bicolor* | [30] |  |
| *Sc*Glu | 3AIU | Q9FYS3 | *Secale cereale* | [31] |  |
| *Ta*Glu1b | 2DGA | Q1XH05 | *Triticum aestivum* | [32] |  |
| *Th*Bgl | 5BWF | A3FPG4 | *Trichoderma harzanium* | [33] |  |
| *Th*Bgl2 | 5JBO | A0A0F9XM91 | *Trichoderma harzianum* | [34] |  |
| *Tr*Bgl | 3AHY | O93785 | *Trichoderma reesei* | [21] |  |
| *Zm*Glu1 | 1E1E | P49235 | *Zea mays* | [35] |  |

**Supplementary Table 1. Bgl library.** The in-house Bgl screening library and their corresponding published crystal structure.

# **Supplementary Figures**

**Supplementary Figure 1.** **In vivo degradation of MANT-N-glucose.** The MANT-N-glucose conversion over time when cultured with E. ramulus DSM15684, B. ovatus DSM2096, or in CFA media without inoculation. Statistical significance determined using a two-way ANOVA test. *p<0.05; ns, not significant; MANT-Glc, MANT-N-glucose.

**Supplementary Figure 2.** **In vitro degradation of MANT-N-glucose.** MANT abundance in the reaction mixtures containing lysates of E. coli expressing a Bgl after overnight incubation with MANT-N-glucose. Abundance values were normalised to the control (lysate of WT E. coli).

## **Supplementary Figure 3. Chromatograms of the *Zm*Glu1-catalysed hydrolysis.** Chromatograms of reaction mixtures containing MANT-*N*-glucose with either no enzyme (black, left column) or purified *Zm*Glu1 (pink, right column) at (A) pH 6.0, (B) pH 7.0, (C) pH 8.0, (D) pH 9.0, and (E) pH 10.0. The peak at RT ~ 2.6 minutes was previously identified as the demethylated MANT-*N*-glucose, anthranilate *N*-glucose [11].

**Supplementary Figure 4. *Zm*Glu1:MANT-*N*-glucose complex structures.** (A-E) The position of MANT-*N*-glucose in the predicted *Zm*Glu1:MANT-*N*-glucose complex across the five ranks (A, rank 1; E, rank 5). (F) The top-ranked *Zm*Glu1:MANT-*N*-glucose complex prediction superimposed with DIMBOA-glucoside from PDB, 1E56 [43]. Structures predicted using Chai-1 [16].
